# Influence of Alkaline Earth Metals on Structure Formation and Magnesium Alloy Properties

**DOI:** 10.3390/ma15124341

**Published:** 2022-06-20

**Authors:** Vadym Shalomeev, Galyna Tabunshchyk, Viktor Greshta, Marek Nykiel, Kinga Korniejenko

**Affiliations:** 1 National University Zaporizhzhya Polytechnik, 64 Zhukovs’kogo Street, 69063 Zaporizhzhya, Ukraine; greshtaviktor@gmail.com; 2 Faculty of Materials Engineering and Physics, Cracow University of Technology, Jana Pawła II 37, 31-864 Cracow, Poland; marek.nykiel@pk.edu.pl (M.N.); kinga.korniejenko@pk.edu.pl (K.K.)

**Keywords:** magnesium alloy, alkali earth metal, intermetallic, modification, heat resistance, micro grain, mechanical property

## Abstract

The main aim of this work is to improve the structure and properties of the magnesium alloy ML5 by modifying it with alkaline earth metals (ALM). The separate and joint influence of calcium and barium on the macrostructure and microstructure of the alloy of Mg-Al-Zn system was investigated. The qualitative and quantitative estimation of the structural components was carried out. Alkali earth metals were included in complex intermetallic phases and serve as additional crystallization centers. Modification of magnesium alloys with alkaline earth metals is established in an amount of 0.05 to 0.1 wt. % increased the bulk percentage of intermetallic phases by ~1.5 times, shifting them towards smaller size groups while simultaneously forming spherical intermetallic phases located in the grain centre and serving as additional crystallization centers. In this case, grain size reduction and significant refinement of the alloy structural components were provided. The dependency of the separate and joint influence of alkali earth metals on the castings complex of properties of the magnesium alloy has been established. Thus, a separate modification of the ML5 alloy provided the maximum level of its strength and ductility with the addition of 0.1% Ca or Ba. The modification of the complex (0.1% Ca + 0.1% Ba) of the magnesium alloy decreased the dimensions of its structural components 1.5 times and increased the strength of the alloy by 20%, the ductility by 2 times and the long-term heat resistance 1.5 times due to the formation of the intermetallic phases of the complex composition. Linear dependences were obtained that describe the influence of the characteristics of the structural components of the modified magnesium alloy on its mechanical properties. The developed technology for modifying cast magnesium alloys with alkaline earth elements provides an improvement in casting quality and allows the reliability and durability of responsible casting operation.

## 1. Introduction

The development and application of new alloys with a larger complex of mechanical and special properties for various industries is a promising field [1,2,3,4]. Magnesium alloys are of great interest in mechanical engineering as lightweight structural materials [5,6], the use of which reduces the weight of structures and vehicles, providing a reduction in fuel consumption and improvement of their dynamic characteristics [7,8,9]. It led to extensive use in the aviation and space industry [6,10].

Castings made of magnesium alloys of the Mg-Al-Zn system have a number of requirements, one of which is the low cost of alloys. This excludes the use of expensive and scarce rare-earth metals (Nd, La, Y) as alloying additives that have been shown to be effective in hardening magnesium alloys at ordinary and elevated temperatures [11,12,13,14,15,16]. Among the alkaline earth metals: beryllium (Be), magnesium (Mg), calcium (Ca), strontium (Sr), barium (Ba), and radium (Ra), the most promising for the modification of magnesium alloys is Ca [6,17]. Ca helps to control the metallurgy of the alloy by increasing grain refinement [6,17]. Large studies were conducted in its addition to magnesium alloys for biomedical applications [18,19], including some new applications in 3D printing technology [20]. In the case of Be, the main influence is the limitation of surface oxidation during casting and welding [21,22]. Other elements have not been investigated in binary systems. Limited research was conducted for Sr and Ba in higher systems as additives for magnesium alloys [23,24].

The analysis of state diagrams of double systems of calcium and barium with magnesium has shown that the interaction of these components may result in the formation of new phases in equilibrium with the solid solution. These phases have higher melting points comparable to the Mg_17_Al_12_ phase in equilibrium with the double solid solution in the Mg-Al system and can contribute to increasing the refractory strength of the alloy [25,26,27,28]. However, it is worth stressing that the influence of the elements listed above is not well understood, because there is a limited amount of research [29,30]. It should also be noted that for this reason magnesium alloys containing alkali earth metals have not yet found wide practical application in mechanical engineering [29,30,31,32]. Therefore, the development of low-cost magnesium alloys with improved mechanical properties and heat resistance, which contain alkali earth metals, is an important task. 

In this research, there are answers to this research gap. The effect of calcium and barium on the structure and properties of castings of magnesium alloys has been studied. The mechanical properties and microstructure of the Mg-Al-Zn system (8.8% Al, 0.35% Mn, 0.32% Zn, 0.01% Fe, 0.007% Cu, 0.02% Si) were investigated.

## 2. Materials and Methods

### 2.1. Materials

The magnesium alloys were melted in an IPM-500 type induction crucible furnace according to serial technology. The melt was refined with flux VI-2 in a dispensing furnace with additional batch sampling of the metal, in which increasing additives of ligatures containing calcium and barium were introduced and sand-clay molds were poured to obtain standard samples with a working diameter of 12 mm. A magnesium alloy without additives was tested simultaneously. 

Samples for mechanical tests were heat treated in Bellevue and PAP-4M furnaces under the following conditions: heating to 415 ± 5 °C, holding for 15 h, air cooling in air and ageing at 200 ± 5 °C, holding for 8 h, air cooling. 

Separate and combined influence of calcium and barium in an amount of 0.05; 0.1; and 1.0 wt. % on the structure and properties of castings from the Mg-Al-Zn alloy system were studied.

### 2.2. Research Methods

The tensile strength and relative elongation of the samples were determined on a P5 tensile test machine at room temperature. The longitudinal strength at elevated temperature was determined on the rupture machine AIMA 5-2 in samples with a working diameter of 5 mm. The microhardness of the structural components of an alloy was defined on a ‘Buehler’ microhardness meter at loading 0.1 N. 

The microstructure of the castings was studied by light microscopy (Neophot 32, Carl Zeiss, Jena, Germany) on thermally treated samples after etching with a reagent consisting of 1% nitric acid, 20% acetic acid, 19% distilled water, and 60% ethylene glycol. The fractographic analysis of the samples fractures was carried out on an electronic scanning microscope ‘JSM-6360LA’. Phase analysis of structural components of magnesium alloys was studied on an electron microscope-microanalyzer with REMMA 202M and REM 16I.

## 3. Results

### 3.1. Microstructure Characteristic

**Figure 1:**
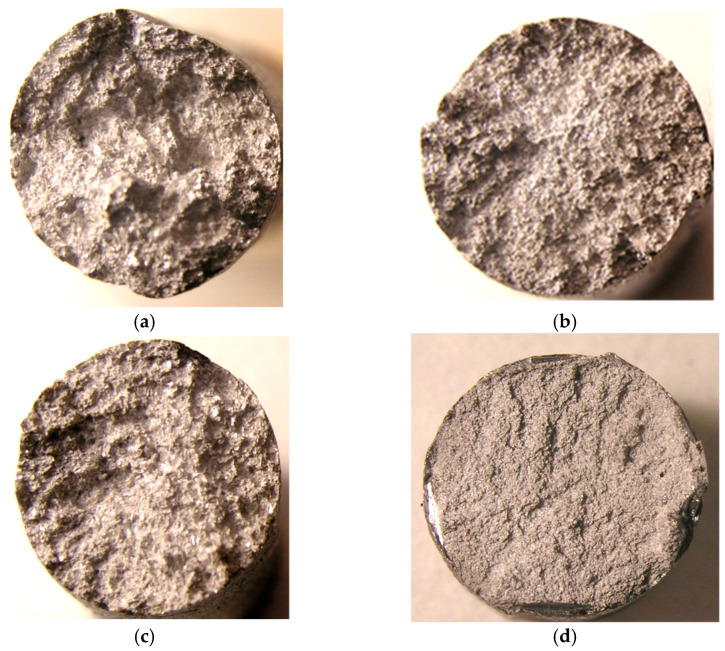
Fracture macrofractograms of ML5 alloy specimens with NFM, ×5: (**a**) without modification; (**b**) with 0.1% Ca; (**c**) with 0.1% Ba; (**d**) with 0.1% Ca + 0.1% Ba.

The macrofractographic study of fractures from ML5 standard alloy has shown the presence of a coarse crystalline structure in the structure. Separate introduction of increasing calcium (Ca) and barium (Ba) additives into the alloy under study refined the cast structure of the metal. At the same time, their combined effect increased the crushing effect and the character of the fracture became a matte fine crystalline (Figure 1).

**Figure 2:**
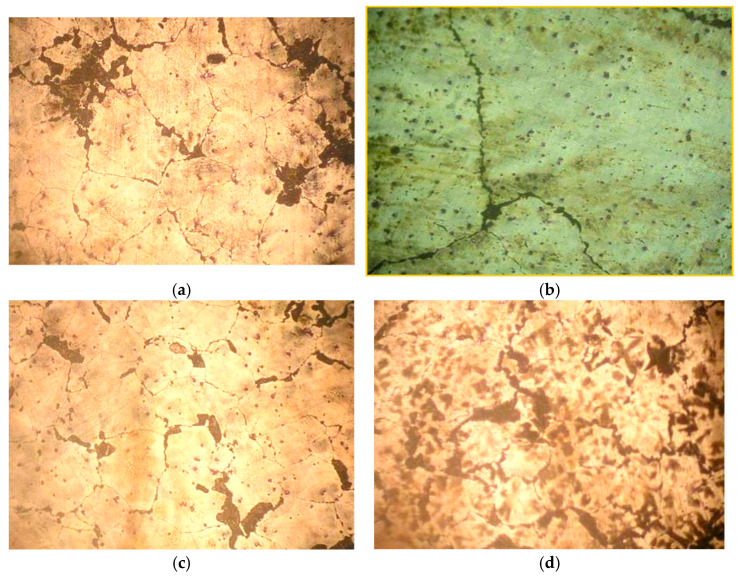
Microstructure of samples of heat treated magnesium alloys, 200: (**a**) without modification; (**b**) with 0.1% Ca; (**c**) with 0.1% Ba; (**d**) with 0.1% Ca + 0.1% Ba.

The microstructure of the standard composition was δ-solid solution with the presence of δ + γ eutectics along the grain boundaries and individual γ-phase intermetallics. The introduction of Ca and Ba into the alloy refined its micrograins. At the same time, the addition of Ba contributed to greater grain than with calcium. The joint modification of the ML5 alloy with NKM contributed to the formation of finer grains (Figure 2).

Increase in the additives of Ca and Ba contributed to the decrease in the second order axis spacing and dendritic cell size (Table 1). Heat treatment increased the homogeneity of the structure and the microhardness of the matrix. As the amount of calcium in the metal increased, the microhardness of the matrix increased, while barium additives decreased its microhardness. Therefore, the microhardness of the eutectics was 1.4 above the hardness values of a matrix δ-solid solution.

**Figure 3:**
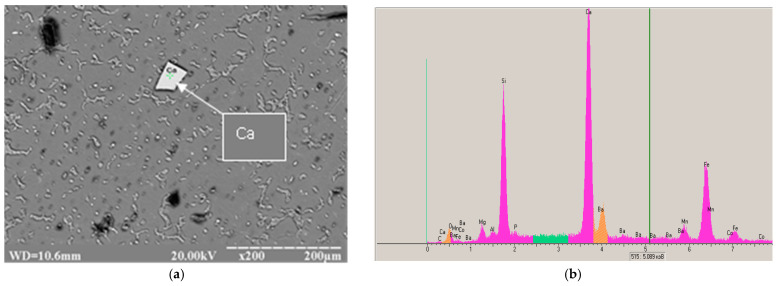
Results of micro-X-ray spectrum analysis of intermetallic phase in ML5 alloy modified with 0.1% Ca + 0.1% Ba: (**a**) Site of analysis; (**b**) Spectrograms of the analyzed sites—Figure 3a.

The micro X-ray diffraction analysis of the ML5 alloy with alkaline ash cenospheres showed that it included include inclusions containing Si, Fe and other impurities (Figure 3 and Table 2). These inclusions, located inside the grain, could be additional crystallization centers that refine it.

It should be noted that barium additives in an amount of 1.0% contributed to the formation of films and led to an uneven distribution of the intermetallic phase in the structure. When calcium was introduced up to 1.0% into the alloy under study, no film formation was detected in the metal.

### 3.2. Numerical Metallographic Analysis

The numerical metallographic analysis of the alloys studied showed the presence of lamellar and spherical intermetallic phases in the metal structure. The lamellar intermetallides were predominantly located at the grain boundaries, and the spherical intermetallides were located in the center of the grain. Spherical intermetallic phases could serve as additional crystallization centers and contribute to grain refinement and structural components of the alloy. By quantitative metallographic analysis, it was established that increasing the content of the alloying elements in the alloy increased the volumetric percentage of the intermetallic phase (Table 3).

The content of calcium and barium in an amount between 0.05 and 0.1% increased more intensively the volume percentage of spherical intermetallides located in the center of the grains, in comparison with lamellar intermetallides. With an increase in the alkali metal content in the alloy of 1.0%, the tendency to increase this parameter prevailed for lamellar intermetallides. This redistribution of the intermetallides contributed to some increase in the ductility of the alloys studied because of grain refinement. The increase in the total number of intermetallides contributed to an increase in the strength of the metal.

Analysis of intermetallide distribution in size groups showed that in the initial magnesium alloy, most lamellar intermetallides were in the size group between 2 and 15 µm. Spherical intermetallides were additionally represented by the size group <2.0 µm. In the magnesium alloy, the investigated modifiers refine the intermetallic phase, and its distribution shifted toward the smaller size groups. At the same time, increasing the element content in the alloy increased the volumetric percentage of intermetallics smaller than 2 μm and decreased the volumetric fraction of large intermetallics larger than 11.6 μm. 

The strength and heat resistance of the examined magnesium alloys increased with an increase in the bulk percentage of intermetallides. Intermetallic compounds located both in the center of the grain and at the grain boundaries hardened the alloy and increased its heat resistance. At the same time, the alloy hardening was more influenced by spherical intermetallics of smaller size groups, which makes them preferable for the formation of the fine-grain structure of the alloy and its hardening.

Ba, which was not dissolved in the solid solution, strongly increased the ductility of the magnesium alloy and had little effect on its strength. Ca was part of the solid solution and increased the strength of the alloy with a slight increase in ductility. The combined modification of these elements increased both the strength and the ductility of the alloy (Table 4).

**Figure 4:**
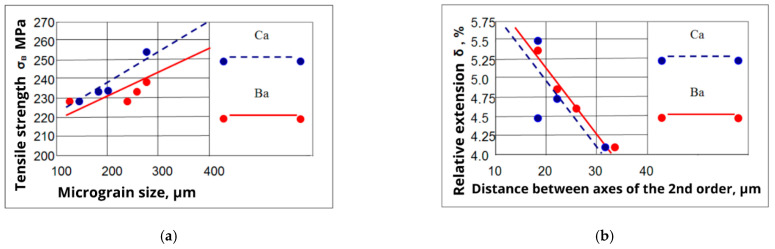
Dependence of strength (**a**) and ductility (**b**) on the size and distance between dendrite axes of the second order for the ML5 alloy modified with calcium and barium.

The linear dependences of the mechanical properties of the ML5 alloy have been constructed from the characteristics of its structural components (Figure 4). 

The strength characteristics of the alloy increase with an increase in the size of micrograins, and the ductility of the alloy increases with a decrease in the distance between the axes of second-order dendrites. The equations describing the effect of micrograin size (d) and the distance between dendrite axes of second order (K) on the ultimate strength and relative elongation in the alloy ML5 with calcium (1, 2) and barium (3, 4) additives have been obtained:

σ_B_ = 238.5 + 0.03*[d], [MPa]; r = 0.02, (1)

δ = 5.86 − 0.05*[K], [%]; r = −0.65,(2)

σ_B_ = 218.4 + 0.096*[d], [MPa]; r = 0.99, (3)

δ = 4.75 − 0.04*[K], [%]; r = −0.53,(4)

The heat resistance properties of the investigated alloys with Ca and Ba were higher than those of the initial alloy. Higher heat resistance was obtained in samples with 0.1% Ca and Ba each. At the same time, their combined effect increased this effect. A further increase in the content of modifiers reduced the long-term strength of the alloy at elevated temperatures. Therefore, the best combination of properties of the magnesium alloy was achieved by modifying it with 0.1% Ca + 0.1% Ba.

## 4. Discussion

The provided investigation shows the possibility of successful modification of magnesium alloys with alkaline earth metals in an amount of 0.05 to 0.1% by weight. Previous literature research on Ca and Ba modification was mainly related to coating application [33,34], including improved corrosion inhibition [35]. Some research related to casting technology also showed improvement in creep resistance for Ba and Ca additives [36,37]. 

The modification described in this article allows the bulk percentage of intermetallic phases to increase by ~1.5 times, shifting them toward smaller size groups while simultaneously forming spherical intermetallic phases located in the grain center and serving as additional crystallization centers. The microstructure obtained from the material is consistent with the predictions and previous analysis made by other authors [38,39].

In the case of the provided research, a reduction in grain size and a significant refinement of structural components were also provided. Moreover, the dependence of the separate and joint influence of alkali-earth metals on the castings complex of properties of the magnesium alloy has been established. Thus, a separate modification of the ML5 alloy provided the maximum level of its strength and ductility with the addition of 0.1% Ca or Ba. It is also consistent with previous works [38,39].

The results show that the modification of the complex (0.1% Ca + 0.1% Ba) of the magnesium alloy decreased the dimensions of its structural components 1.5 times and increased the strength of the alloy by 20%, ductility by 2 times and long-term heat resistance by 1.5 times due to the formation of the intermetallic phases of the complex composition. In addition, linear dependences were obtained describing the influence of the characteristics of the structural components of the modified magnesium alloy on its mechanical properties. These improvements can be useful for the industrial applications of magnesium alloys in casting technology. Furthermore, it is worth saying that the additives used are relatively inexpensive solutions compared to modification using, for example, rare elements such as yttrium or scandium [6,40,41].

The results obtained allow the development of a technology for modifying cast magnesium alloys with alkaline earth elements, which improves the quality of castings and allows the increase of reliability and durability of responsible casting operations. This result may be useful for application of this material in aviation, automotive, and other industries [23,42].

## 5. Conclusions

A positive effect of the modification of magnesium alloys with alkaline earth metals on the structure formation and properties of the metal has been established. Additives of calcium and barium in the alloy from 0.05 to 1.0 wt. % contributed to the refinement of its macro- and microstructure up to ~40%, as well as an increase in mechanical properties and heat resistance.

- It is shown that microalloying of magnesium alloys from 0.05 to 0.1 by weight leads to an increase in the volume content of intermetallic compounds, their grinding and spheroidization.
- Linear dependences of the mechanical properties of an alloy of the Mg-Al-Zn system on the characteristics of its structural components have been obtained. Equations are constructed that describe the influence of the micrograins size and the distance between the axes of dendrites of the 2nd order on the ultimate strength and relative elongation of a magnesium alloy modified with alkaline earth metals.

## Figures and Tables

**Table 1:** Average dimensions of structural components and microhardness in magnesium alloy samples with Ca and Ba.

Content, wt. % (Calculated)	Micrograin Size, Microns	Distance between Axes of 2nd Order Dendrites, µm	Matrix Micro-Hardness HV, MPa	
			Before Heat Treatment	After Heat Treatment
—	140	21	1115.9	1227.1
0.05% Ca	130	19	1126.3	1234.1
0.10% Ca	120	18	1135.4	1267.1
1.0% Ca	130	19	1186.8	1283.8
0.05% Ba	120	18	1085.7	1110.8
0.10% Ba	110	17	1034.8	1042.0
1.00% Ba	100	16	988.7	1012.6
0.10% Ca + 0.10% Ba	80	15	1132.6	1264.5

**Table 2:** Chemical composition of the intermetallic phase in the ML5 alloy modified with 0.1% Ca + 0.1% Ba; the site marked in Figure 3a (mass %).

Element	Ca	Ba	Fe	Si	Mn	Mg	Total
Mass %	36.67	0.86	28.98	24.87	3.58	2.84	100

**Table 3:** Volumetric percentage of intermetallic compounds and their distribution by size group in the magnesium alloy with Ca and Ba.

Element	Content, %	Intermetallide Distribution (V- 10-3, %) by Dimensional Groups, μm						
		<2	2…3.9	4…7.9	8…11.5	11.6…15	15.1…19	Total
Standard		6/0	18/54	36/30	30/12	30/12	18/0	138/108
Ca	0.05 0.1 1.0	42/0 66/0 78/78	84/72 90/150 162/126	24/42 42/42 42/24	12/24 12/18 6/6	30/0 18/0 18/0	18/0 6/0 0/0	210/138 234/210 306/234
Ba	0.05 0.1 1.0	48/0 90/18 150/57	48/108 30/114 24/114	30/42 48/42 72/30	24/36 18/30 18/24	36/6 18/6 12/6	18/0 18/0 6/0	204/192 222/210 282/231

Note: The numerator is the volumetric percentage of lamellar intermetallides and the denominator is the spherical ones.

**Table 4:** Mechanical properties and heat resistance (average values) of magnesium alloy with Ca and Ba.

Contents Elements, wt. %	Mechanical Properties				$\tau_{150}^{80}$ Hour
	Without Heat Treatment		After Heat Treatment		
	σ_B_, MPa	δ, %	σ_B_, MPa	δ, %	
—	160.8	2.5	228.4	3.2	141.6
0.05% Ca	167.0	2.8	235.4	3.4	147.9
0.10% Ca	177.0	2.6	246.0	3.7	208.4
1.0% Ca	188.0	3.0	253.0	3.5	190.0
0.05% Ba	161.0	3.6	236.0	3.8	145.4
0.10% Ba	160.9	4.2	237.0	4.6	177.2
1.0% Ba	163.0	4.8	230.0	5.3	151.2
0.1% Ca + 0.1% Ba	185.8	4.7	250.5	5.1	210.5

## Data Availability

Not applicable.
